# Mr. Pursh on the Eupatorium Perfoliatum

**Published:** 1812-06

**Authors:** Frederick Pursh


*To the Editors of the Medical and Physical Journal.*

GENTLEMEN,

THE plant, of which I observe some account in your last Journal, is undoubtedly the *Eupatorium perfoliatum* of Linnæus, or Thorough-wort; and more commonly Bone-set of the inhabitants of North America.

It seems to have been known to the Indians of America long before the Europeans were made acquainted with its medicinal properties, as most of those Indians, even in the remotest part of the country, know the plant well, and occasionally use it as one of their chief remedies in violent colds and fevers.

The oldest European planters of Virginia and other western parts of the United States, informed me, that this plant had been known to their parents very far back, as a medicine in diseases above mentioned; and most people are acquainted with its virtues in different diseases, principally those supposed to be occasioned by violent colds.

So far I only state from tradition; but the following facts I have from my own experience.

In the year 1808, while I was on a botanical journey through the western parts of the United States, a disease prevailed over the country, known by the name of influenza. The neighbourhood of Onondago, near Lake Ontario, where I then chiefly resided, was particularly afflicted with it; and the lake fever, very similar to the yellow fever in its effects, had made its appearance in a most alarming shape. Persons attacked with the influenza, and afterwards catching the lake fever, died in a few days; nay, in some cases, in a few hours; and not one instance has come to my knowledge, where, both diseases united, medical aid had proved successful, though men of decided skill were not wanting in that part of the country.

Among the rest of sufferers by the influenza, I had the misfortune to be one. My pursuits in natural history not allowing me to take the necessary care of myself, and being on an excursion to Lake Ontario in very rainy and rough weather, on my return I fell sick, and to my utmost alarm it proved to be the lake fever.

If not too tedious, I shall take the liberty to decribe, as far as recollection will admit, the beginning and end of this frightful disease. I then lodged at the house of John Adams, of Onondago-hollow, and, the morning after my return from Oswego, I found myself unwell, or as I thought very much fatigued, and laid longer than usual in bed: the people people of the house called me to breakfast, which was quite unusual to them and me. I endeavoured to get down stairs, but was not able to taste any thing. A head ach, which I am not able to describe, but the pain of which I still recollect with horror, obliged me to return to my chamber, still hoping that rest would restore me to my usual health; but, instead of this, I scarcely had reached my bed when I fell in a high fever, the head ach increasing to a still higher degree, visions of different descriptions came before my eyes, till at last I lost all my senses, and cannot recollect what my feelings or situation had been for about three hours. When I recollected myself again, I considered the danger I should be in if I suffered a relapse of this fever, ruminating in my mind what to do in such a desperate case. The *Eupatorium perfoliatum* came into my recollection; and, having heard so much of its powerful effects in similar cases, I was determined to give it a trial. Accordingly, I endeavored to get up, and, notwithstanding my weakness, I undertook to walk about a mile in search of the plant. On my return, I was taken several times by faintings, and had to sit down on the road. However I got home with a good quantity of the plant in flower. I ordered a strong infusion of the plant (bruised) with gin, and, going to bed again, as soon as the liquor had drawn well, I began to take a wine-glass full every ten or fifteen minutes. I began with it about two o'clock in the afternoon, and kept on at that rate for about sixteen hours; and, strange it must appear, that, during that time, though more than two pints of gin had been used, I did not feel, with all the weakness I was in, the least intoxicating effect of the liquor.

About six next morning, vomiting and stools to an amazing degree took place; and, for about two hours, matter of a most offensive smell was discharged both ways. About eight I found myself somewhat easier, and after covering myself well, I fell in a natural sound sleep. When I awoke, I found myself in an excessive sweat; the bed clothes were ringing wet, but my violent head ach had ceased, and I was, the weakness excepted, quite well. I ventured to get up, took some soup, and used my infusion during the day in a more diluted state, continuing it for about thirty-six hours. I was thus completely cured of the lake fever, and a few days recovered me completely.

My example made most of the people of Onondago, who were afflicted with the influenza, use the *Eupatorium*, and they universally found relief from it. Since that time, I have been informed, it has been used in the lake fever by professional men, and always with the wished-for effect.

A few

A few years after this I had the fever and ague, and, though going through a regular course of cinchona bark, I had so many relapses of it, that I was obliged to take my old friend *Eupatorium* again; and found it more efficacious than all the barks I had taken.

A number of other people troubled with the ague, have been relieved by my method of taking *Eupatorium*; but, as it is of very disagreeable taste, it requires resolution to take enough of it, for in small doses only no good can be done. Here I must observe, that, taken by a person in health, it will occasion vomiting in a very small dose; but, if fever matter* rests on the stomach, it requires a great quantity to operate; but, if taken until it has the effect, relief certainly will follow.

These few observations are thrown together in a hasty manner, but it will be highly gratifying to me if they should tend to promote an investigation of this interesting plant. I have no doubt that observations and experiments made by skilful professional men, will find in the *Eupatorium perfoliatum* a very valuable article to be added to the materia medica.

With the highest esteem, I have the honor to be,

Gentlemen,

Your obedient and humble Servant,

FREDERICK PURSH.

* Mr. Pursh, though a highly accomplished botanist, is not of the medical profession; and this will account and apologise for some modes of expression not strictly in the language of the science. We have let the term *fever matter* remain, because it is expressive of a peculiar fact, that of the stomach, when in a morbid state, admitting a quantity of this active vegetable to be taken into it, not only with safety but advantage; which quantity, in a state of healthy action, would, perhaps, destroy life. We cannot let this note pass without expressing our obligation to Mr. Pursh for the valuable facts he has communicated, and which we hope will stimulate the profession to a further inquiry.—EDITORS.

